# Cardiovascular and Renal Effects of Bromocriptine in Diabetic Patients with Stage 4 Chronic Kidney Disease

**DOI:** 10.1155/2013/104059

**Published:** 2013-08-04

**Authors:** Oliva Mejía-Rodríguez, Jorge E. Herrera-Abarca, Guillermo Ceballos-Reyes, Marcela Avila-Diaz, Carmen Prado-Uribe, Francisco Belio-Caro, Antonio Salinas-González, Helios Vega-Gomez, Cleto Alvarez-Aguilar, Bengt Lindholm, Elvia García-López, Ramón Paniagua

**Affiliations:** ^1^Coordinación de Educación e Investigación en Salud, Unidad de Medicina Familiar No. 80, Instituto Mexicano del Seguro Social (IMSS), Morelia, Mich, Mexico; ^2^Escuela Superior de Medicina, Instituto Politécnico Nacional, México, DF, Mexico; ^3^Unidad de Investigación en Enfermedades Nefrológicas (IMSS), México, DF, Mexico; ^4^Hospital General Regional No 1. (IMSS), Morelia, Mich, Mexico; ^5^Divisions of Baxter Novum and Renal Medicine, Karolinska Institutet, Stockholm, Sweden

## Abstract

*Objective*. The objective of this study was to investigate the effect of bromocriptine (BEC) on left ventricular mass index (LVMI) and residual renal function (RRF) in chronic kidney disease (CKD) patients with type 2 diabetes (T2D). *Research Design and Methods*. A 6-month double-blind randomized controlled trial was conducted in 28 patients with T2D and stage 4 CKD with increased LVMI. Fourteen patients received BEC (2.5 mg, initially 1 tablet with subsequent increase to three times a day) and 14 received a placebo (PBO; initially 1 tablet with subsequent increase to three times a day). Cardiovascular changes were assessed by monitoring 24 h ambulatory blood pressure, two-dimensional-guided M-mode echocardiography, and N-terminal brain natriuretic peptide (NT-proBNP) plasma levels. RRF was evaluated by creatinine clearance and cystatin-C plasma levels. *Results*. Both BEC and PBO groups decreased blood pressure—but the effect was more pronounced in the BEC group. Average 24 h, diurnal and nocturnal blood pressures, and circadian profile showed improved values compared to the PBO group; LVMI decreased by 14% in BEC and increased by 8% in PBO group. NT-proBNP decreased in BEC (0.54 ± 0.15 to 0.32 ± 0.17 pg/mL) and increased in PBO (0.37 ± 0.15 to 0.64 ± 0.17 pg/mL). Creatinine clearance did not change in the BEC group and decreased in the PBO group. *Conclusions*. BEC resulted in a decrease on blood pressure and LVMI. BEC also prevented the progression of CKD while maintaining the creatinine clearance unchanged.

## 1. Introduction

Cardiovascular diseases (CVD) are the most frequent cause of morbidity and mortality in patients with chronic kidney disease (CKD) [1, 2]. High blood pressure and left ventricular hypertrophy (LVH) associated with volume overload [3–5] are two of the earliest and most frequent cardiovascular disorders. These conditions are independent risk factors for death and loss of residual renal function (RRF) [6–9]. 

Adequate control of fluid overload is a key point in the prevention and handling of blood pressure and LVH. Nevertheless, in predialysis patients, when salt and fluid restriction is not sufficient, the need arises for pharmacological support. Although there are several classes of antihypertensive drugs available, the use of renin-angiotensin-aldosterone system (RAAS) blockers is considered the most effective renal and cardioprotective treatment [10, 11]. 

CKD is characterized by an increase in sympathetic activity, which appears to be triggered by the unhealthy kidney [12–14], with elevated plasma levels of epinephrine (E) and nor-epinephrine (NE) [15, 16], as well as a reduction in dopaminergic activity, as reflected by an increase in prolactin (PRL) plasma levels [17]. Sympathetic overactivity and circulating NE are two important promoters of cardiovascular disease through their vasoconstrictive action and stimulation of the hypertrophy of cardiomyocytes and cardiac fibrosis [18–20]. Bromocriptine (BEC; bromoergocriptine), a type-2 dopamine receptor (D2) agonist, can reduce sympathetic activity and levels of circulating NE and thus contribute to the management of high blood pressure and LVH in patients with CKD. In previous studies, it has been demonstrated that the use of low doses of BEC permits adequate blood pressure control and reduces LV mass in both peritoneal dialysis and hemodialysis patients [21–23]. 

In addition to its cardioprotective effects, BEC also has important metabolic effects, reducing insulin resistance and improving glycemic control in overweight and patients with T2D. Quick-release BEC has been accepted by the US Food and Drug Administration for the treatment of T2D [24–26]. Taking these facts into consideration, BEC seems to have advantages in metabolic and blood pressure control for patients with T2D and advanced stages of CKD. The aim of this study was to address the hypothesis that BEC ameliorates high blood pressure and LVH, and delays the loss of RRF in patients with T2D and stage 4 CKD. 

## 2. Materials and Methods

### 2.1. Design

 A single-center, double-blind randomized controlled trial (RCT) was conducted in diabetic patients with stage 4 CKD. The protocol was approved by the scientific and ethics boards of the hospital and was performed according to the Helsinki Declaration. The protocol was also registered in the Australian New Zealand Clinical Trials Registry (ANZCTR; number ACTRN12610000779077; http://www.ANZCTR.org.au/ACTRN12610000779077.aspx). All patients signed a written informed consent form before beginning the study. 

### 2.2. Patients

 Prevalent CKD patients from the out-patient clinic for diabetes and hypertension were invited to participate. Inclusion criteria were age 18–80 years, blood pressure ≥140/90 mmHg or on antihypertensive therapy, creatinine clearance (CrCl) ≤30 mL/minute, and LV mass index >116 gm^2^ in men and >104 gm^2^ in women. Patients were excluded if they were receiving dopamine D2 receptor antagonist treatment or if they had had metabolic complications in the last 3 months, heart failure, cancer, or positivity for HIV. Antihypertensive medication at baseline (number of patients in BEC versus PBO) was calcium channel blockers (CCB; 14 versus 12), *α*-1 blocker (prazosin; 3 versus 1), furosemide (13 versus 10), thiazides (1 versus 0), and spironolactone (0 versus 1).

### 2.3. Procedures

Patients underwent an initial baseline evaluation, demographic and clinical data, measurements of biochemical parameters, glomerular filtration rate (GFR), echocardiography, and 24-hour ambulatory blood pressure were recorded. Patients were then randomized to receive an initial dose of 2.5 mg a day (tablet) of bromocriptine mesylate (BEC), or placebo (1 tablet) with a subsequent increase to 2 tablets per day after one week and 3 tablets from the third week. Patients were followed each week during the first four weeks and every month thereafter, for a total of 6 months. Antihypertensive drug doses were adjusted when necessary. Patients were prescribed a diet containing less than 100 mmol/day of sodium and reduced content of phosphorus according to current clinical guidelines.

### 2.4. Biochemistry

Overnight-fasting blood samples were taken following 30 minutes of complete rest in recumbent position from the antecubital vein without stasis between 7:00 and 8:00 a.m. at baseline and at each visit. Samples were centrifuged, and plasma and serum were separated and kept at −70°C until assay. 24-hour urine collection was obtained for urinary volume and creatinine clearance (CrCl) analysis. Blood glucose, urea, creatinine, cholesterol, and triglycerides were measured by routine, standard techniques in automatic equipment. Cystatin-C levels were measured by immunoturbidimetric technique (N-Latex Cystatin C, Dade Behring, Germany). Prolactin (PROL-CTK4, DiaSorin Inc., Saluggia-Vercelli, Italy), aldosterone (ALDO-CTK2, DiaSorin Inc., Saluggia-Vercelli, Italy), and NT-proBNP (Diagnostic Automation, Calabasas, CA, USA) were measured in plasma by commercial RIA kits. GFR was calculated using the cystatin-C values and validated formulas [27]. 

### 2.5. Echocardiography

Echocardiography was performed in M mode and bidimensional mode by the same blinded cardiologist (echocardiography ATL, model HDI 3500, Bothell, WA, USA) and sectorial transducer (Model VIBS 24 OA, SSA) following recommendations of the American Society of Cardiology. 

### 2.6. Ambulatory Blood Pressure

24-hour systolic blood pressure (SBP), diastolic blood pressure (DBP), mean arterial pressure (MAP), and heart rate (HR) were assessed using a Space Labs 90207 recorder (SpaceLabs Inc., Redmond, WA, USA). Recording began at 7:00 a.m. and ended at 7:00 a.m. the following day. The monitor was programmed to measure the blood pressure every 30 minutes during the day and night.

### 2.7. Statistics

Data are presented as mean ± SE for continuous variables and percentage for discontinuous variables. Data were analyzed as an intention-to-treat analysis. Differences between groups were analyzed by independent Student *t*-test; changes intragroup in the studied variables along the time were analyzed with a general linear model for repeated measures. To determine if the effects on LVM and renal function of BEC were independent of blood pressure and metabolic control an ANCOVA analysis was performed. Statistical significance was considered with a *P* value <0.05. The data were processed with SPSS 18.0 (SPSS, Chicago, IL, USA).

## 3. Results

Thirty-two patients were initially included in the study. Of these, two declined to participate in the study and two started peritoneal dialysis just after the deadline for the initial evaluation. Fourteen patients from each group were included in the final analysis. 

Table 1 shows relevant data at baseline; there were no differences between the intervention (BEC) group and the placebo (PBO) group, indicating successful randomization. 

### 3.1. Cardiovascular Effects

**Figure 1:**
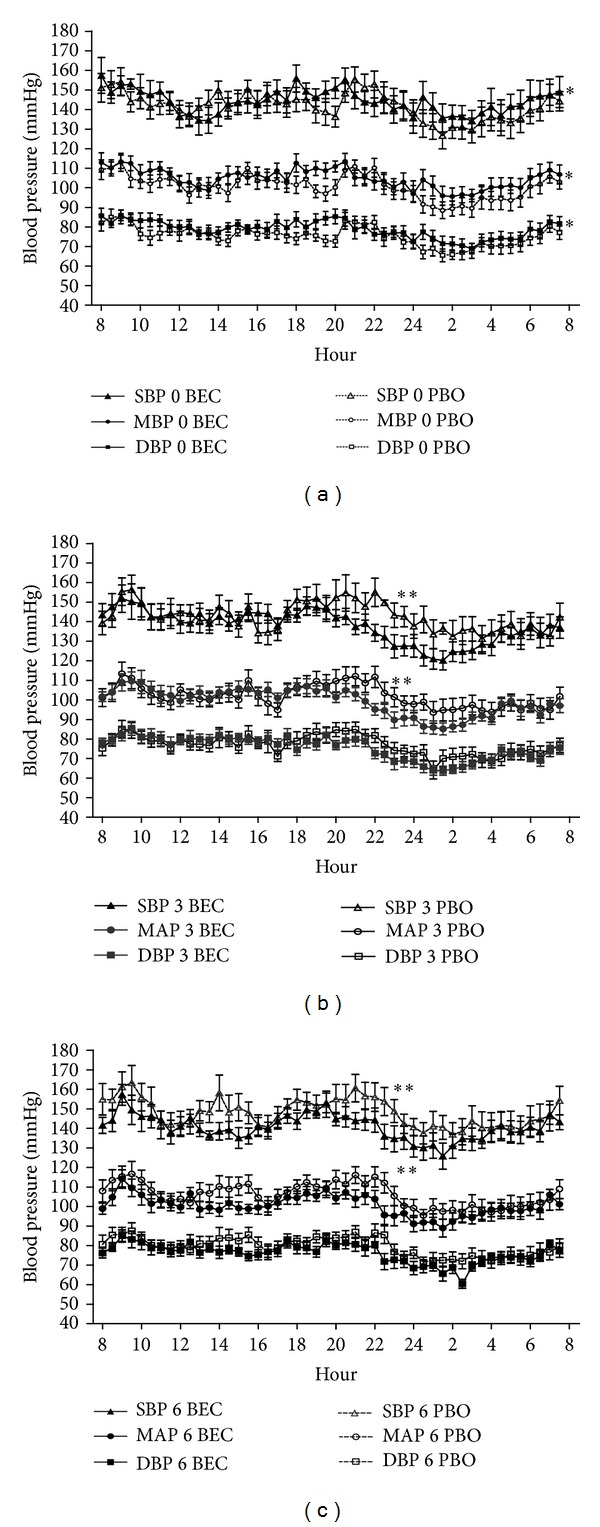
24-hour systolic (SBP), diastolic (DBP), and mean arterial (MAP) ambulatory blood pressure in patients treated with bromocriptine (BEC) and placebo (PBO). The 24-hour ABP values are presented for each group at baseline (a), 3 months (b) and 6 months (c). There were no differences between groups at baseline, but, at 3 and 6 months, significant reductions were seen in the BEC group, both during diurnal and nocturnal hours. *P < 0.05.

*24-Hour Ambulatory Blood Pressure*. Twenty-four hour ambulatory blood pressure profiles were similar in both groups at baseline and showed loss of circadian rhythm, Figure 1(a). Significant reductions in 24-h ABP profiles were observed in the BEC group at 3 and 6 months, Figures 1(b) and 1(c). In this group, circadian rhythms were recovered, showing a significant reduction during nocturnal hours as compared with diurnal hours. In the PBO group, SBP was reduced but at a different level than the BEC, and this group did not recover circadian rhythm. No differences were observed in HR at any time point. In the BEC group, two patients required an extra dose of channel calcium blockers (CCB) and one prazocin; in two patients the dose of CCB was decreased; whereas in the PBO group an extra dose of CCB was required in two patients and additional medication was needed in 8 patients (CCB in one, prazosin in 5, hydralazine in 2, and thiazides in 1) only in one patient the furosemide dose was reduced.

### 3.2. Echocardiographic Parameters

**Figure 2:**
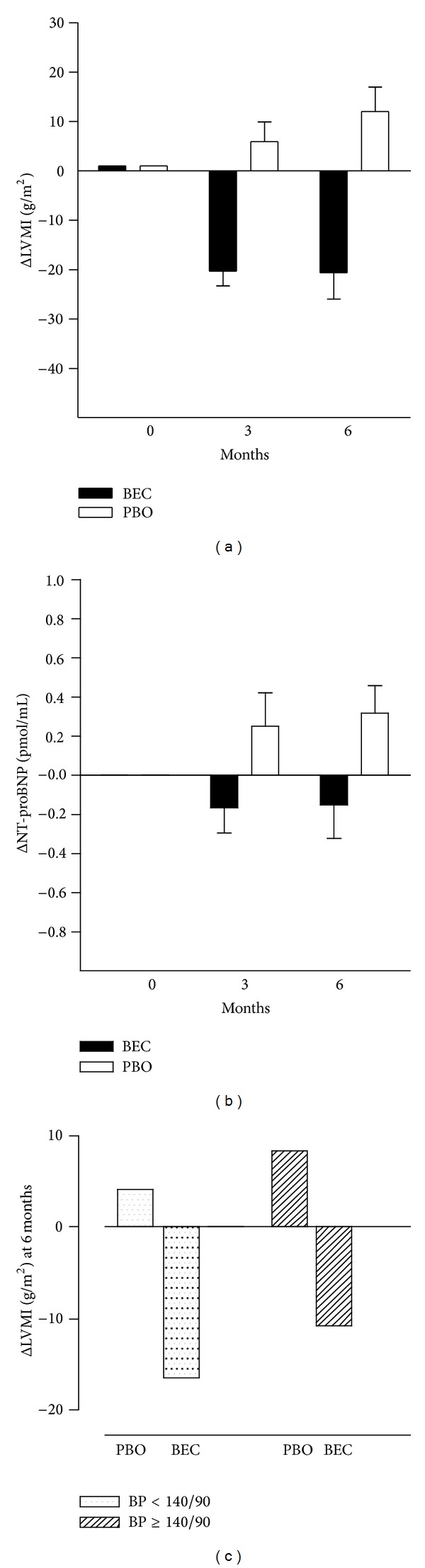
Changes in left ventricular mass index (LVMI; (a)) and N-terminal brain natriuretic peptide (NT-proBNP; (b)) in patients treated with bromocriptine (BEC) and placebo (PBO). LVMI decreased in the BEC group and increased in the PBO group. A similar pattern of changes was observed for NT-proBNP which decreased in the BEC group and increased in the PBO group. Changes in left ventricle mass index (Δ LVMI; (c)) were more significant in BEC than in PBO group independently if blood pressure was within (<140/90 mmHg) or not within normal limits.

Patients in the BEC group showed significant reductions in LV mass and interventricular wall thickness after 3 and 6 months of treatment, whereas in patients in the PBO group these parameters tended to increase, resulting in significant differences between the groups at 3 and 6 months, Table 2. The decrease in LVMI in the BEC group was accompanied by a decrease in the levels of NT-proBNP while the opposite was true for the PBO group, Figures 2(a) and 2(b). Nine patients in the BEC group showed echocardiographic values within normal limits at the end of the study, whereas in the PBO group no patient reached this target. LVMI is highly dependent on (blood pressure) BP, to a better analysis of changes of LVMI, patients of both groups were categorized according to control of BP. Among the patients in the four groups, BEC with BP within normal limits, BEC with BP over normal limits, PBO within normal limits, and PBO over normal limits, only the BEC groups had significant decrements in LVMI versus PBO group independently if BP was within or not within normal limits, Figure 2(c). The left ventricle ejection fraction was preserved, and no significant changes were observed in any group, Table 2.

### 3.3. Renal Effects

 In the PBO group, creatinine and cystatin C increased whilst CrCl declined significantly, and GFR calculated by cystatin-C values also tended to decline. In contrast, although there was an observed increase in creatinine and cystatin C, CrCl remained statistically unchanged in the BEC group, Table 2. Two patients from the control group started peritoneal dialysis during the study in the fourth and fifth months respectively.

### 3.4. Biochemical and Variables

Significant reductions in glucose and cholesterol were observed in the BEC group at three months; in the PBO group these parameters increased; at this point the antidiabetic treatment was modified (four patients in the BEC group decreased the hypoglycemic oral medication or insulin dose on the contrary five patients in the control group increased the dose of oral medication or insulin) after this modification the glucose and cholesterol levels decreased in both groups without significant difference between the groups at the end of the study. Hemoglobin levels and hematocrit did not change along the study in any group, Table 2. Small but significant reductions in serum triglyceride values from baseline to 3 and 6 months were seen only in the BEC group. In the BEC group, PRL levels showed significant reduction at 3 and 6 months in comparison with baseline values, and PRL levels were also significantly lower as compared with those in the PBO group, in which PRL levels instead showed significant increments at the end of follow-up, Table 2. 

## 4. Discussion

Data from this randomized controlled trial can be summarized as follows: oral administration of BEC for 6 months in patients with T2D and CKD resulted in: reduced blood pressure, restored circadian rhythm of blood pressure, reduced LV mass, delayed decline of RRF, and improved metabolic control. 

The results of the current study support the importance of targeting the overactivity of the sympathetic system and increased levels of NE in patients with CKD as suggested by previous reports from clinical and experimental studies [12–19]. High NE plasma levels, sympathetic overactivity, and hyperprolactinemia have been recognized in different clinical conditions, including obesity, insulin resistance, hypertension, and CKD, suggesting a reduced dopaminergic tone [28–31]. Furthermore, it has been proposed that the association between low-birth weight and the development of hypertension and metabolic syndrome in later life could be due to a reduction in dopaminergic tone and an increase in sympathetic activity [32, 33]. Therefore, it seems plausible that the use of dopamine agonists in these conditions could be of benefit for the patients, and this is supported by several studies demonstrating beneficial effects of BEC on cardiovascular and metabolic parameters [21–26, 34]. In the current study, blood pressure was better controlled in the patients receiving BEC with minimal changes in the antihypertensive medication. Furthermore, PRL levels decreased significantly as compared with the PBO group, suggesting an improvement in the dopaminergic tone. A cardioprotective effect of BEC, expressed as a reduction in blood pressure, has been previously reported in hypertensive patients with end-stage renal disease (ESRD) on peritoneal dialysis or hemodialysis [17, 21–23]. More recent reports suggest that the elevated levels of NE and the sympathetic overactivity stimulate and promote cardiomyocyte hypertrophy and myocardial fibrosis resulting in increased LV mass [19, 20, 35]. In a group of patients with ESRD on continuous ambulatory peritoneal dialysis treated with BEC, we showed a reduction in LV mass assessed by echocardiography and reduction in NT-proBNP levels, confirming a cardioprotective effect of BEC [22]. The cardioprotective effect was also observed in a randomized clinical trial in which the authors reported a reduction of cardiovascular risk in patients taking BEC compared with PBO [36]. High blood pressure is probably the strongest stimuli for increasing LVMI, for further clarification of the BEC effect, patients in both groups were divided according to the control of BP (cut-point 140/90). We found the BEC effect to be independent of BP control. BEC administration seems to have additive effect with BP values within control limits on LVMI.

The nephroprotective effect of BEC with better preservation of renal function as measured by CrCl was observed in the current study; the pathophysiological pathways that explain this effect have not been sufficiently explored, and we cannot assure that there are some other independent factors not directly related to the heart or the kidney. However, there are some experimental studies reporting that circulating levels of PRL are elevated in an NZB/NZW murine model of systemic lupus erythematous (SLE) and that the treatment with BEC prevents or retards the development of lupus nephropathy in mice [35, 37, 38]. On the other hand, high plasma PRL levels have also been found in humans with SLE and are associated with episodes of SLE reactivation and progression of lupus nephropathy [39]. The effect of BEC has been attributed to suppression of PRL secretion, preventing the proinflammatory action of the hormone. We did not monitor inflammation markers and are thus unable to prove whether or not this mechanism alone or in combination with better blood pressure control is the basis for the observed effects of BEC including its apparent beneficial impact on preservation of renal function. 

Previous studies, including some RCT's testing the effect of quick-release BEC formulations, have shown a beneficial effect of BEC in the control of glycaemia and glycated hemoglobin levels, and the frequency of serious adverse events and cardiovascular events was lower in the BEC treated group when compared with placebo controls [24–26, 40]. In the current study, a better control of glycaemia, cholesterol, and triglycerides levels was observed in patients in the BEC group. The mechanism by which BEC improves metabolic control in diabetic patients is currently under investigation. Some central effects have been mentioned, but the peripheral effect in reducing sympathetic tone and a possible antiinflammatory action may also be involved [24–26].

Some limitations of the current study should be noted. Only surrogate markers of clinical outcomes were monitored; a longer follow-up period analyzing all-cause and cardiovascular mortality in a larger group of patients is needed to provide definitive proof of the beneficial effects of BEC. The study enrolled a small number of patients; however, significant results were still obtained with an acceptable level of confidence. 

In conclusion, the results of this RCT show that BEC treatment of stage 4 CKD patients resulted in reduction of LV mass, improved blood pressure control, and lowered NT-proBNP and PRL levels; it also prevented the decline of RRF, and improved the metabolic control. These promising results deserve to be tested in a more open setting resembling the day-to-day conditions to be validated. Additional studies are needed to clarify the pathophysiological mechanisms involved that are regulated by BEC. From a cost-effectiveness point of view, BEC should be advantageous as it is cheaper than most of the common drugs currently used as cardio- and renal protection.

## 5. Summary 

 To test previously reported benefits of BEC for metabolic control in patients with type 2 diabetes and for reducing left ventricular mass in CKD, an RCT was conducted for six months in patients with stage 4 CKD and increased LV left ventricular mass. The main findings include a better control of blood pressure, measured by 24-hour ambulatory monitoring. Echocardiography showed significant reduction in left ventricular mass, paralleled by a reduction in NT-proBNP levels. BEC also improved blood glucose and lipid control and lowered PRL levels. No significant adverse events of BEC were observed. 

## Figures and Tables

**Table 1:** Demographic and clinical data at baseline.

Variable	Bromocriptine	Placebo	*P* value
*N*	14	14	
Gender (M/F)	6/8	6/8	
Age (years)	61.7 ± 8.8	60.4 ± 7.7	NS
Evolution time of diabetes (years)	18.3 ± 2.1	17.4 ± 1.6	NS
Evolution time of hypertension (years)	8.9 ± 3.0	7.1 ± 1.1	NS
Weight (kg)	67 ± 4	64 ± 3	NS
Height (m)	1.6 ± 0.1	1.6 ± 0.1	NS
BMI (kg/m^2^)	26.8 ± 1.2	25.9 ± 1.5	NS
SBP (mmHg)	177 ± 20	174 ± 22	NS
DBP (mmHg)	101 ± 8	99 ± 7	NS
Heart rate (beats/minute)	80.7 ± 6.7	80.0 ± 2.2	NS
LVMI (g/m^2^)	143.9 ± 23.1	146.2 ± 27.6	NS
CrCl (mL/min)	16.8 ± 7.7	17.7 ± 4.3	NS
Glucose (mmol/L)	8.16 ± 0.95	7.23 ± 0.93	NS
Cholesterol (mmol/L)	5.65 ± 0.33	5.82 ± 0.48	NS
Triglycerides (mmol/L)	2.39 ± 0.22	2.25 ± 0.32	NS
Hemoglobin (mmol/L)	7.24 ± 0.24	7.14 ± 0.24	NS
Hematocrit (%)	34.98 ± 1.04	34.92 ± 1.19	NS

SBP: systolic blood pressure; DBP: diastolic blood pressure; LVMI: left ventricular mass index; CrCl: creatinine clearance.

**Table 2:** Echocardiography and biochemical parameters in patients receiving bromocriptine (BEC) or placebo (PBO).

Variable	Baseline		3 months		6 months		*P* value
	BEC	PBO	BEC	PBO	BEC	PBO	
Echocardiography data							
LVM (g)	241.9 ± 15.5	237.6 ± 16.3	207.1 ± 14.0	248.6 ± 16.3	207.6 ± 17.0	260.4 ± 14.4	0.003
LVMI (g/m^2^)	143.9 ± 6.2	146.2 ± 7.4	123.5 ± 5.6	152.2 ± 7.3	123.2 ± 8.8	158.2 ± 6.5	0.001
LVDD (mm)	42.4 ± 1.5	41.1 ± 1.3	41.4 ± 1.4	42.7 ± 1.2	41.4 ± 1.6	43.8 ± 1.2	NS
LVSD (mm)	26.21 ± 4.0	26.6 ± 5.9	26.72 ± 5.1	25.5 ± 6.1	26.22 ± 5.8	24.8 ± 5.8	NS
LV EF (%)	72.5 ± 12.8	73.4 ± 12	71.7 ± 13.1	73.1 ± 13	73.0 ± 11.9	75.4 ± 11	NS
LVPWT (mm)	14.7 ± 0.4	13.6 ± 0.5	13.2 ± 0.5	13.8 ± 0.4	13.2 ± 0.5	14.2 ± 0.4	0.042
IVST (mm)	14.1 ± 0.4	13.6 ± 0.5	13.6 ± 0.5	13.8 ± 0.4	13.1 ± 0.3	14.2 ± 0.4	0.001
Biochemical variables							
S-Cr (mmol/L)	288.88 ± 17.6	280.22 ± 17.6	291.72 ± .26.5	322.6 ± 73.3	309.4 ± 26.5	362.4 ± 17.6	0.01
CrCl (mL/min)	16.85 ± 7.71	17.75 ± 4.34	17.06 ± 8.79	14.30 ± 3.81	17.53 ± 10.37	11.89 ± 3.58	0.03
Cystatin C (mg/L)	3.03 ± 0.95	3.03 ± 0.74	3.05 ± 0.95	3.43 ± 0.96	3.58 ± 1.1	3.48 ± 0.84	0.05
GFR_cyst_ (mL/min)	30.1 ± 11.0	30.3 ± 8.2	29.3 ± 7.9	28.0 ± 6.3	29.4 ± 14.3	26.9 ± 4.5	NS
Glucose (mmol/L)	8.16 ± .0.95	7.23 ± 0.93	6.50 ± 0.66.	7.65 ± 0.16	5.83 ± 0.38	5.88 ± 0.44	NS
Cholesterol (mmol/L)	5.65 ± 0.33	5.82 ± 0.48	4.85 ± 0.33	5.48 ± 0.38	4.53 ± 0.33	5.18 ± 0.33	0.01
Triglycerides (mmol/L)	2.39 ± 0.22	2.25 ± 0.32	2.10 ± 0.22	2.06 ± 0.22	2.14 ± 0.28	2.21 ± 0.28	NS
Prolactin (mmol/L)	7.4 ± 3.2	3.6 ± 0.4	5.0 ± 2.5	4.4 ± 0.7	3.3 ± 1.1	6.8 ± 1.5	0.026
Aldosterone (mmol/L)	485 ± 319	577 ± 557	594 ± 402	594 ± 446	586 ± 427	510 ± 369	NS
NT-proBNP (pmol/mL)	0.5429 ± 0.74	0.3743 ± 0.34	0.3771 ± 0.34	0.6257 ± 1.05	0.3914 ± 0.20	0.6921 ± 0.90	0.01

LVM: left ventricle mass; LVMI: left ventricle mass index; LVDD: left ventricle diastolic diameter; LVSD: left ventricle systolic diameter; LV EF: left ventricle ejection fraction; LVPWT: left ventricle posterior wall thickness; IVST: interventricular septum thickness. S-Cr: serum creatinine; CrCl: creatinine clearance; GFR_cyst_: glomerular filtration rate calculated by serum cystatin C.
